# Re-fixation Using the Belt Loop Technique for Postoperative Subluxation of a Multifocal Intraocular Lens After Retinal Detachment Surgery: A Case Report

**DOI:** 10.7759/cureus.79585

**Published:** 2025-02-24

**Authors:** Takashi Nagamoto, Norimitsu Ban, Hirohisa Kubono, Kotaro Suzuki, Kazuno Negishi

**Affiliations:** 1 Department of Ophthalmology, Keio University School of Medicine, Tokyo, JPN; 2 Department of Ophthalmology, Keiyu Hospital, Yokohama, JPN; 3 Department of Ophthalmology, Tokyo Saiseikai Central Hospital, Tokyo, JPN

**Keywords:** belt loop technique, dislocation, flanged intrascleral fixation, multifocal intraocular lens repositioning, needle externalization approach, prolene, subluxation

## Abstract

Intraocular lenses (IOLs) have evolved significantly with the development of cataract surgery. Today multifocal intraocular lenses (MFIOLs) are increasingly used due to their ability to reduce dependence on glasses and improve patient satisfaction. However, late complications, such as IOL dislocation, pose challenges, especially for patients wishing to retain MFIOLs. The belt loop technique (BLT), a sutureless scleral fixation method, has been proposed as a simple and minimally invasive solution for IOL repositioning. Despite its potential, clinical evidence supporting BLT, particularly its needle externalization approach, remains limited. This report describes a 64-year-old male with MFIOL dislocation. Following a history of macular-off rhegmatogenous retinal detachment (RRD) repair by vitrectomy with gas tamponade, the patient experienced vision decline due to IOL displacement. Using the BLT needle externalization approach, both haptics of the MFIOL were successfully repositioned. Postoperatively, the patient achieved stable and improved near and distance visual acuity without significant glare or halos, maintaining these outcomes over a half year. This case demonstrates the clinical efficacy of the BLT needle externalization approach for MFIOL repositioning. The technique offers a minimally invasive, effective alternative for managing dislocated IOLs, even in complex cases involving MFIOLs. This method holds promise as a valuable addition to the repertoire of IOL fixation techniques, while the long-term stability requires further study.

## Introduction

Cataract is the leading causative disease of visual impairment worldwide, and surgery is the common curative treatment [1]. Intraocular lenses (IOLs) have evolved significantly with the development of cataract surgery. Early lenses were monofocal, but today premium IOLs such as multifocal intraocular lenses (MFIOLs) are available. MFIOLs are a treatment option that can improve patient satisfaction by reducing dependence on glasses, and the cumulative number of surgeries is increasing [2].

On the other hand, late complications of cataract surgery include IOL subluxation and dislocation [3], which may lead to more patients requesting premium IOLs, including MFIOL re-fixation. The belt loop technique (BLT), an IOL sutureless intrascleral fixation technique presented by McCabe, is a simple re-fixation method. BLT is available as an intraocular or extraocular method and can be applied to multifocal IOLs [4]. However, a PubMed® search on December 4, 2024, using the keyword “Belt Loop Technique” revealed only reports on flange strength in cadaveric eyes [5], and no report on its clinical usefulness in living subjects was found.

In this case report, we describe a case of MFIOL subluxation after macular-off rhegmatogenous retinal detachment (RRD) fixed by the BLT needle externalization approach to bilateral haptics, in which good visual acuity was obtained.

## Case presentation

Case report

**Figure 1:**
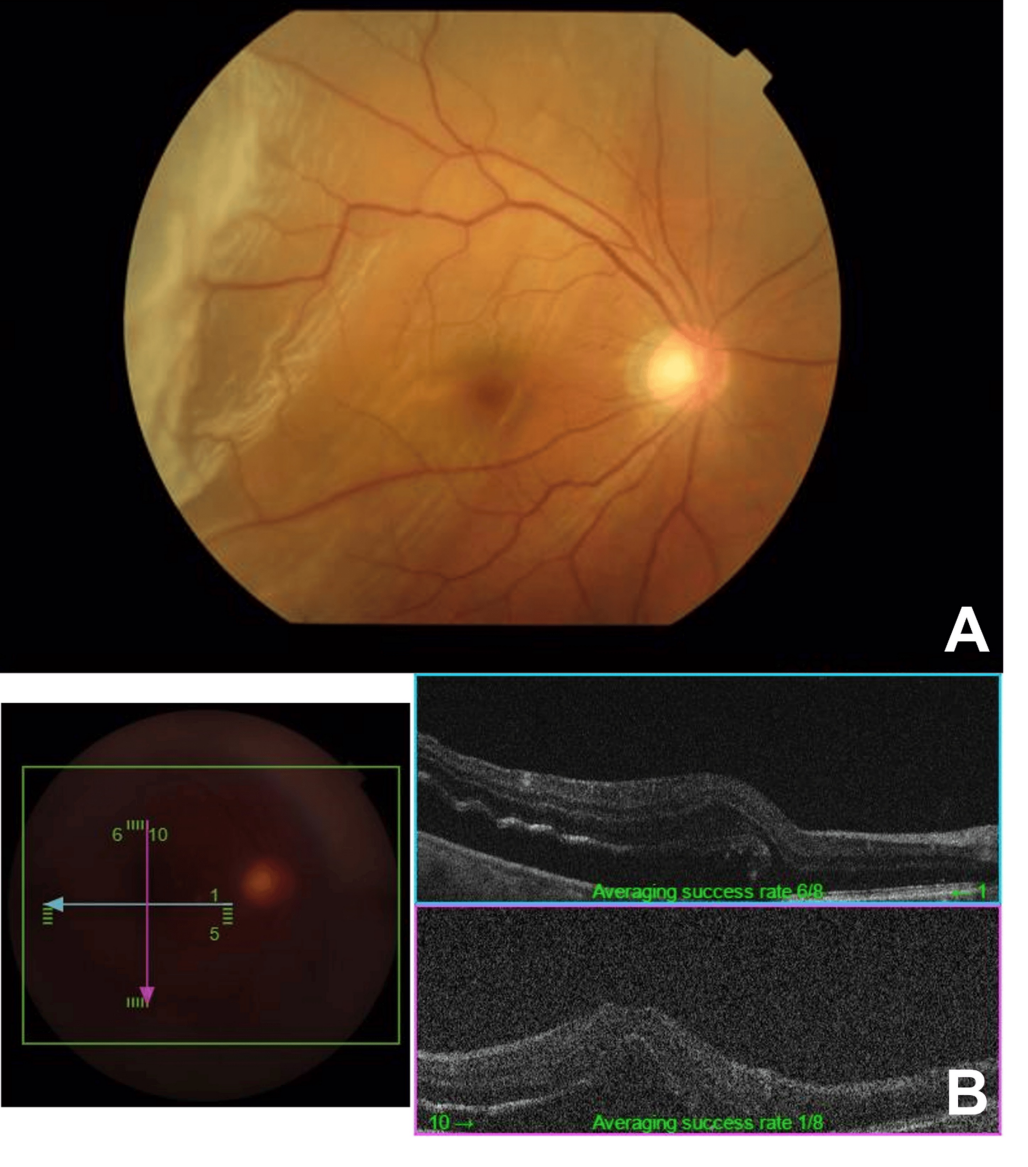
Macular-off rhegmatogenous retinal detachment in the right eye. Fundus photograph (A) and optical coherence tomography (B).

**Figure 2:**
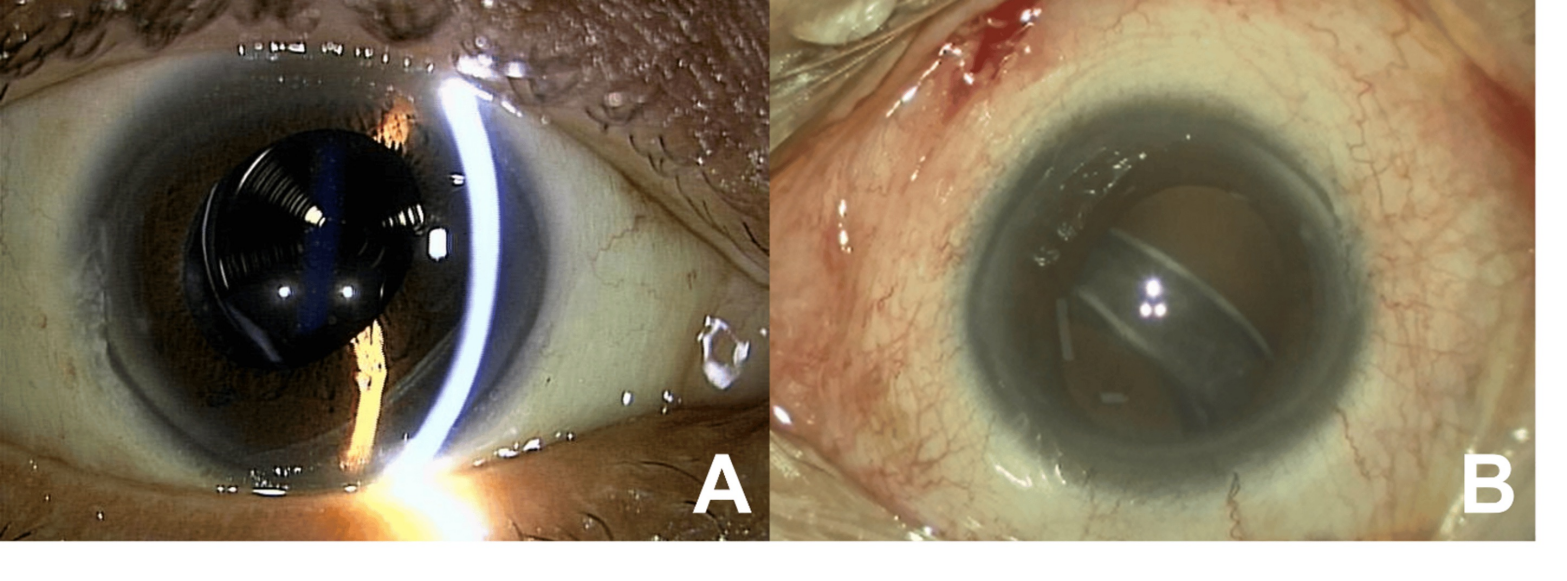
Subluxation of an intraocular lens observed one month after retinal detachment surgery. Although it was difficult to see with a slit lamp (A), the lens was displaced downward when the patient was in the supine position (B). Zonular dialysis was seen at least 270°; other than the nasal upper side, the lower haptics were completely off.

**Figure 3:**
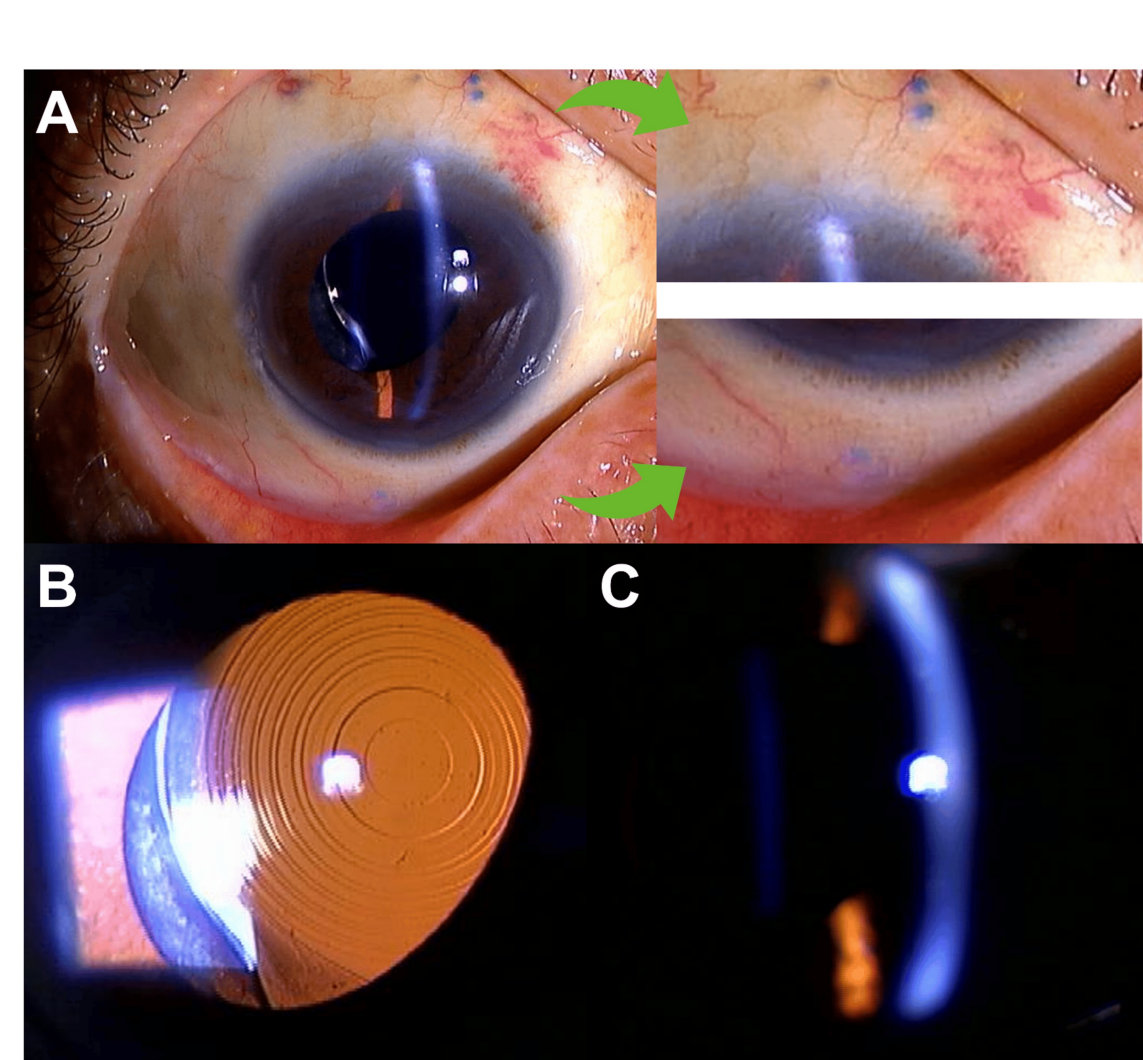
Anterior segment photographs taken one month after the belt loop technique surgery. Overall view and enlarged view of the flange (A). The centering of the IOL was good (B) and no significant tilt of the IOL was observed (C). IOL: intraocular lens

A 64-year-old male underwent bilateral cataract surgery in the year 2021 at the previous hospital. He was inserted TECNIS Synergy® (Johnson & Johnson Vision, Santa Ana, CA, USA) +12.0D in the right eye, Intensity (Hanita Lenses, Kibbutz Hanita, Israel) +12.5D in the left eye, and capsular tension rings in both eyes. The IOLs in both eyes were low power, suggesting the long axial length and high risk of retinal detachment and IOL dislocation. In October 2023, the patient visited our hospital because he became aware of visual field abnormality in the right eye five days before and sudden vision loss in the same eye the day before. A macular-off RRD caused by 11 o'clock multiple retinal tears of the right eye was observed (Figure 1). The patient’s uncorrected distance visual acuity (UCVA) had decreased to 0.2, and his best-corrected visual acuity (BCVA) was 0.3 p with a refraction of -0.50 D cylinder at 85° in decimal visual acuity. A vitrectomy of the right eye was performed on the day of presentation. By November 2023, his BCVA had recovered to 1.2, with a refraction of +0.75 D sphere. In December 2023, the patient complained of decreased visual acuity in the supine position. His BCVA had decreased to 1.0 p, with a refraction of +0.50 D sphere, and IOL subluxation was observed (Figure 2). Because of the patient's preference for MFIOL preservation, the BLT externalization procedure was performed in January 2024. In February 2024 (Figure 3), BCVA improved from 1.0 p, with a refraction of +0.50 D sphere preoperatively to 1.2 with a refraction of +1.00 D sphere, -0.25 D cylinder at 95°. Uncorrected near (at 30 cm) visual acuity (UCNVA) was also good at 0.9 p and best-corrected near (at 30 cm) visual acuity (BCNVA) was 1.0 p with a refraction of +0.75 D sphere. In July 2024, the refractive values were stable. UCVA was 1.0 and BCVA was 1.2 with a refraction of +1.00 D sphere, -0.25 D cylinder at 95°. UCNVA was 1.0, BCNVA was 1.2 with a refraction of +1.00 D sphere, -0.25 D cylinder at 95°. No postoperative complications were observed. There was no worsening of glare or halos, and the patient was highly satisfied with the results. Video 1 shows the summary of the case of the BLT needle externalization approach.

**Video 1:** The summary of the case of belt loop technique needle externalization approach.

Belt loop technique needle externalization approach

**Figure 4:**
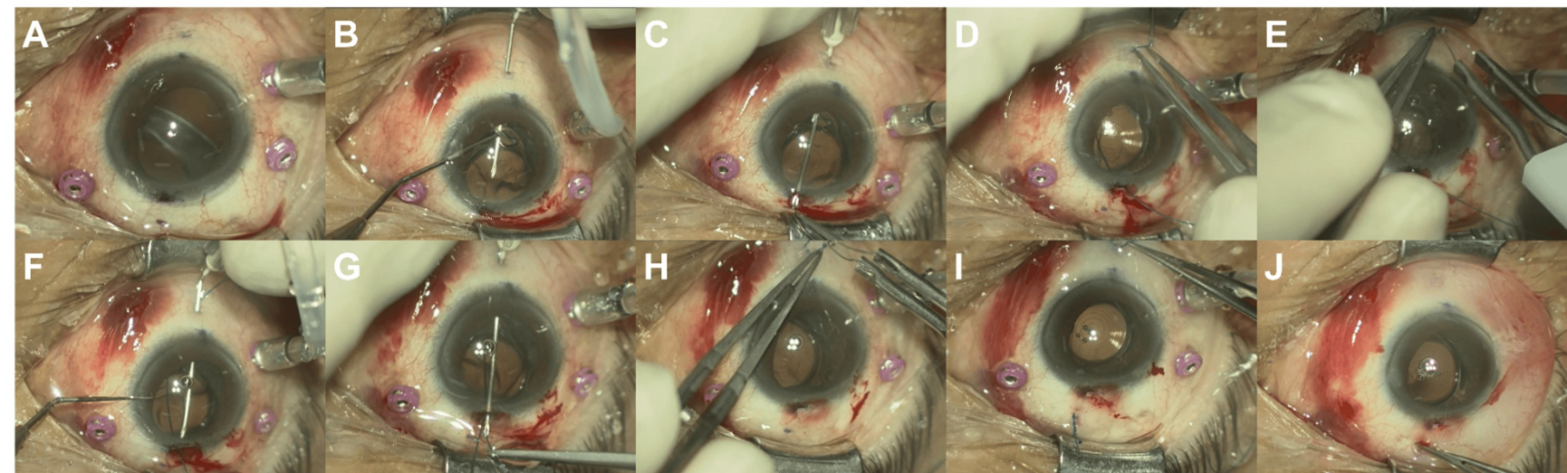
Each procedure of belt loop technique needle externalization approach. Three ports for vitrectomy were placed, and markings depending on the position of zonular dialysis and haptics were made on the eye (A). A bent 27G needle was inserted into the sclera 2 mm from the limbus, and the needle tip passed under the haptics to puncture the lens capsule (B). A 5-0 prolene was inserted into the tip of the needle that was temporarily outside the eye (C). The needle was removed from the sclera, and the suture was passed under the haptics (D). A temporary flange was created with sufficient length (E). A 27G needle was inserted approximately 0.5 mm forward from the initial insertion point, this time passing over the haptics (F). The tip of the needle was pulled out through the paracentesis on the other side. The other side of the 5-0 prolene was inserted into the needle (G). The needle was removed, and another flange was created (H). The contralateral haptic was threaded with 5-0 prolene in the same manner, and the intraocular lens (IOL) position was adjusted to the center (I). The excess sutures were cut, the flanges were recreated, and the IOL was fixed. The flange was buried under the conjunctiva (J).

**Figure 5:**
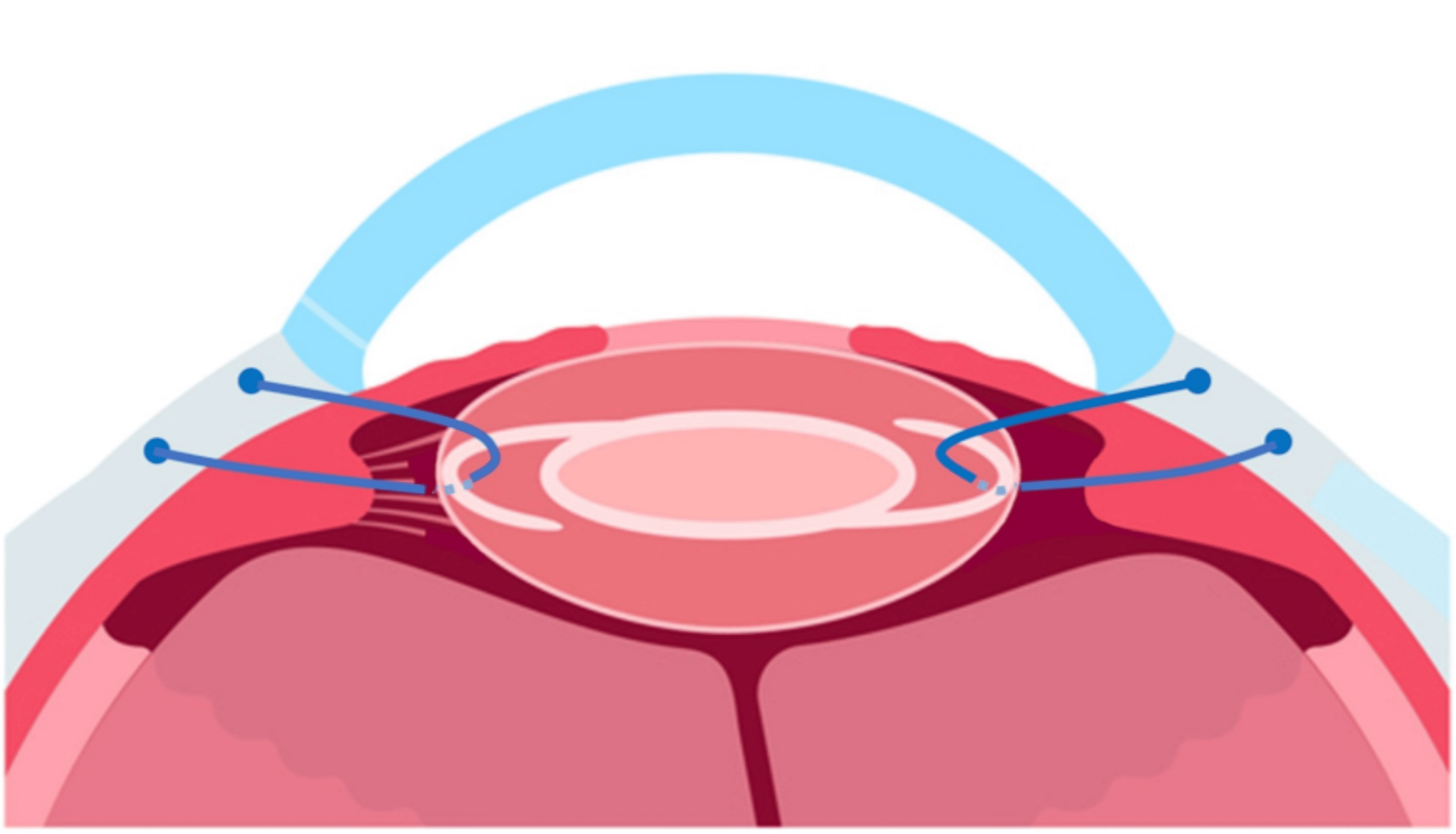
Schematic of in-the-bag intraocular lens using belt loop technique.

We identified and marked the area where the zonules of Zinn were highly damaged and IOL haptics was present at 6.5 o'clock, along with the contralateral side (Figure 4A). A paracentesis was made in the marked area and the cornea at 3 o'clock. A 27G needle (Flowmax 27G (Short Bevel), needle length 19 mm (3/4 inch), Nipro, Japan) bent at the base was inserted into the sclera at 2 mm from the marked corneal limbus, and the needle tip was used to perforate the lens capsule, passing under the haptics (Figure 4B). The needle tip is temporarily placed outside the eye from the contralateral paracentesis. The needle was externalized and a 5-0 prolene was inserted into the needle outside the eye (Figure 4C), the needle was withdrawn from the sclera (Figure 4D) and temporarily fixed with a flange (Figure 4E). Approximately 0.5 mm anterior to the initial puncture point, a 27G needle was inserted using the same procedure, this time over the haptics (Figure 4F), and the needle tip was brought out into the contralateral paracentesis. The contralateral side of the 5-0 prolene was inserted into the needle (Figure 4G), and the needle was withdrawn from the sclera to create another flange (Figure 4H). The other side follows the same procedure to create a “belt loop” around the haptics (Figure 5). We adjusted the position (Figure 4I), recreated the flange, and fixed the lens. The flanges were buried so that they were hidden by the conjunctiva (Figure 4J).

## Discussion

We experienced a case of MFIOL subluxation in which good near and distance visual acuity was achieved by the BLT needle externalization approach in spite of visual deterioration due to macular detachment. To the best of our knowledge, this is the first case report to demonstrate the simplicity of the BLT extraocular method and its favorable visual outcome.

Previously, the suture method proposed by Lewis [6] was the main treatment for IOL displacement, but in recent years Yamane et al. reported a minimally invasive sutureless method [7]. Although this method has the limitation of using a three-piece IOL, McCabe was inspired by Yamane et al. [7] and Canabrava et al. [8] to devise a BLT that could be applied to all intracapsular fixed lenses [9]. Mahmood et al. [10] showed a method that is quite similar to the intraocular BLT method, but it is considered to be a more invasive manipulation because of the conjunctival incision. Assia and Wong reported a case series of 18 cases in which IOLs were fixed using a method similar to the intraocular BLT method with 6-0 polypropylene [11]. However, the actual effectiveness of the method on visual function was still unknown because they did not use the extraocular BLT method as in this case, and the details of their cases were not described. In our opinion, the extraocular method, which offers more freedom, is easier than the intraocular method, which requires a limited space.

In general, decentration of MFIOLs significantly affects optical quality [12], and it is considered unsuitable for IOL re-fixation. Synergy® is prone to unpleasant photopsia [13], and IOL malposition can worsen glare and halo [14], so it was thought that re-fixation would tend to reduce visual function. Furthermore, there is a known association between retinal detachment and IOL displacement [15], eyes with retinal disease are often considered unsuitable for MFIOL implantation [16]. However, in our case, after macular detachment treatment, the visual function was well-maintained, with no significant photic phenomena reported. Our result may suggest that this method is an optimal re-fixation technique.

While most methods of IOL re-fixation [7,8,10,11,17-19] require delicate threading in the limited space of the intraocular, this BLT needle externalization approach is mainly performed outside the eye, where there is more flexibility and it is relatively easy. Because it is a minimally invasive method, it may be used in cases of dislocation of polymethyl methacrylate IOLs, which require large incisions for removal.

Previous reports [18,19] have raised concerns about the cheese-wiring effect, which may affect the long-term stability of lens capsules, so attention should be paid to them when using this method. Although there was no significant change in refraction at six months, the long-term stability of this method may be greatly affected by individual factors such as the degree of fibrosis of the lens capsule, and there are possibilities of iatrogenic ciliary body damage due to this procedure and the occurrence of iritis due to IOL instability. In this case, a mild hyperopic shift may have occurred after the procedure due to the posterior shift of the IOL, although the refractive state before retinal detachment was unknown. This is a single case report of a patient after macular detachment, and it is possible that the low psychological hurdle before BLT surgery led to postoperative satisfaction, so further investigation is needed in a larger number of cases.

## Conclusions

The cumulative number of MFIOL surgeries has been increasing, and there are concerns that the number of MFIOL dislocation cases will increase. Because most of the current scleral fixation methods have restrictions on the types of IOL that can be used and are not compatible with most MFIOLs, there is a need for simplified and less invasive surgical procedures for IOL re-fixation. The BLT needle externalization approach mainly involves external manipulation, offering easier handling and potentially reducing surgical invasiveness compared to previous IOL re-fixation techniques. This method can provide a good prognosis of visual function and would be a minimally invasive treatment option for cases of in-the-bag MFIOL subluxation.
